# Massachusetts Veterinary Association

**Published:** 1897-12

**Authors:** Henry S. Lewis

**Affiliations:** Secretary


Secretary.

### MASSACHUSETTS VETERINARY ASSOCIATION.

The regular monthly meeting of the Association was held at 19 Boylston Place, September 22d. The President, J. F. Winchester, called the meeting to order. Members present were: Drs. Burr, Emerson, Howard, Lee, Lewis, McLaughlin, Parker, Peters, Pierce, Rogers, Sheldon, Stickney, Winslow, and Winchester. The minutes of the previous meeting were adopted as read.

The Executive Committee reported favorably on the application of Dr. D. L. Bolger, of Cambridge, and by vote of the Association he was elected.

Drs. Winchester and Parker were given a vote of thanks for their report of the annual meeting of the United States Veterinary Medical Association.

The subject of a State Veterinary Bill was fully discussed, and Drs. McLaughlin, Howard, and Parker were appointed a committee on legislation.

Several interesting cases were reported.

Dr. Winchester showed two specimens taken from the choroid plexus of the lateral ventricles, which were covered with what looked to be a calcareous deposit. They were given to Dr. Frothingham for analysis.

Dr. Peters reported a case of strangles which, on post-moterm examination, showed the same deposit.

Adjourned at 10.45 P.M.

Henry S. Lewis,
Secretary.

